# Fungal Biotechnology and Application 3.0

**DOI:** 10.3390/jof12060408

**Published:** 2026-06-04

**Authors:** Baojun Xu

**Affiliations:** Food Science and Technology Program, Department of Life Sciences, Beijing Normal-Hong Kong Baptist University, Zhuhai 519087, China; baojunxu@bnbu.edu.cn; Tel.: +86-756-3620636

## 1. Preface

Fungi represent one of the most diverse and functionally versatile groups of organisms on Earth, with profound impacts on human health, food security, industrial manufacturing, environmental remediation, and ecological sustainability. In recent decades, fungal biotechnology has evolved from traditional fermentation practices to cutting-edge interdisciplinary research spanning synthetic biology, multi-omics, material science, and precision medicine. Novel fungal strains, secondary metabolites, enzymatic systems, and mycelium-based materials continue to unlock new applications in cosmetics, food, pharmaceuticals, agriculture, and environmental engineering.

This Special Issue “Fungal Biotechnology and Application 3.0” in the Section of “Fungi in Agriculture and Biotechnology” published in *Journal of Fungi* continues the successful series of Fungal Biotechnology and Application 1.0 and 2.0, aiming to gather frontier advances in fungal biology, biotechnology, and translational applications. It welcomes original research and comprehensive reviews covering beneficial and pathogenic fungi, with a focus on industrial utilization, secondary metabolism, novel strain prospecting, pathogenic mechanisms, fungal ecology, and the cultivation of edible and medicinal mushrooms. After rigorous peer review, this Special Issue has published 13 high-quality papers, including original research and review articles, reflecting the latest progress and trends in fungal biotechnology.

## 2. Overview of Contributions

The 13 publications in this Special Issue cover a broad spectrum of topics, including fungal biocontrol and plant growth promotion, the discovery of bioactive secondary metabolites, the development and functional regulation of edible mushrooms, biodegradation of environmental pollutants, mycelium-based composite materials, lignin degradation and lignocellulose utilization, multi-omics applications, and the health-promoting functions of edible mushrooms.

### 2.1. Fungal Biocontrol and Agricultural Applications

Several studies highlight the value of fungi as sustainable biocontrol agents. Li et al. (2026) reported that *Trichoderma nordicum* V1 exhibits strong antagonism against multiple oomycete pathogens and effectively controls cucumber damping-off and pepper blight while promoting plant growth, supporting its potential as a biopesticide in vegetable production [Contribution 1]. Similarly, Zhou et al. (2025) demonstrated that *Trichoderma brevicompactum* 6311 strongly inhibits *Phytophthora capsici* and enhances pepper growth via induced resistance and growth-promoting metabolites, providing a green strategy for managing *Phytophthora blight* [Contribution 2].

### 2.2. Secondary Metabolites and Drug Discovery

Yuan et al. (2026) established an HSQC–DeepSAT-guided strategy to rapidly discover α-pyrone analogs from endophytic *Diaporthe kyushuensis*, identifying new compounds with selective antifungal activity [Contribution 3]. This work underscores the efficiency of spectrum-based strategies in mining cryptic natural products from fungi. Zhao et al. (2025) reviewed global transcriptional regulator engineering in *Aspergillus*, a powerful approach to activate silent biosynthetic gene clusters and expand the discovery of fungal secondary metabolites [Contribution 4].

### 2.3. Developmental Biology and Multi-Omics of Edible Fungi

Jiang et al. (2025) performed transcriptomic analysis across three developmental stages of *Sarcomyxa edulis*, revealing key genes and pathways regulating fruiting-body formation [Contribution 5]. Im et al. (2024) investigated the effects of light on *Flammulina velutipes*, showing that blue and green light strongly modulated pigmentation and gene expression related to cytochrome P450 and monooxygenases [Contribution 6]. Xie et al. (2025) systematically reviewed genomic, transcriptomic, and proteomic applications in edible fungi, providing a framework for molecular breeding and quality improvement [Contribution 7]. Kumar and Xu (2025) reviewed the chemical profiles and health benefits of *Dictyophora indusiata*, highlighting its polysaccharides, terpenoids, and other bioactives with antioxidant, immunomodulatory, and neuroprotective functions [Contribution 8].

### 2.4. Environmental Biotechnology and Bioremediation

Nóbrega et al. (2025) demonstrated that *Penicillium guaibinense* CBMAI 2758 efficiently biodegradeDs chloroquine, producing less toxic metabolites and offering a green approach for pharmaceutical pollutant remediation [Contribution 9]. Nilza et al. (2024) reported that *Aspergillus ochraceus* DY1 efficiently degraded alkali lignin and lignocellulosic biomass, supporting applications in biofuels, pulp waste treatment, and biorefinery [Contribution 10].

### 2.5. Mycelium-Based Green Materials

Jiménez-Obando et al. (2025) developed insulating mycelium composites using agro-industrial waste and *Ganoderma lucidum*, with low thermal conductivity comparable to commercial insulation [Contribution 11]. Jinanukul et al. (2024) optimized substrate ratios of corn husk and sawdust for *Lentinus sajor-caju* mycelium boards, tailoring mechanical, physical, and sound-absorbing properties for eco-friendly construction and packaging [Contribution 12]. Onomu et al. (2024) reviewed fungal applications in aquaculture, including feed improvement, immunity enhancement, and bioremediation [Contribution 13].

## 3. Outlook

Collectively, the 13 papers in this Special Issue illustrate the vitality and interdisciplinary nature of modern fungal biotechnology. Fungi not only serve as cell factories for food, medicines, and materials, but also provide eco-friendly solutions for disease control, pollution remediation, and carbon neutrality. Future directions include: (1) high-throughput discovery and functional characterization of novel fungal strains and metabolites; (2) the precision engineering of fungal chassis for high-value compound production; (3) the mechanistic elucidation of fungal–host and fungal–environment interactions; (4) the scale-up and commercialization of mycelium materials and biocontrol products; and (5) the integration of multi-omics and artificial intelligence to accelerate fungal biotech innovation.

